# Supplementary material for: Interplay between an ATP-binding cassette F protein and the ribosome from Mycobacterium tuberculosis
Source: Nat Commun. 2022 Jan 21;13:432. doi: 10.1038/s41467-022-28078-1 (PMC8782954; doi:10.1038/s41467-022-28078-1)
Supplement: Supplementary file 1 — Supplementary Information [file 41467_2022_28078_MOESM1_ESM.pdf]

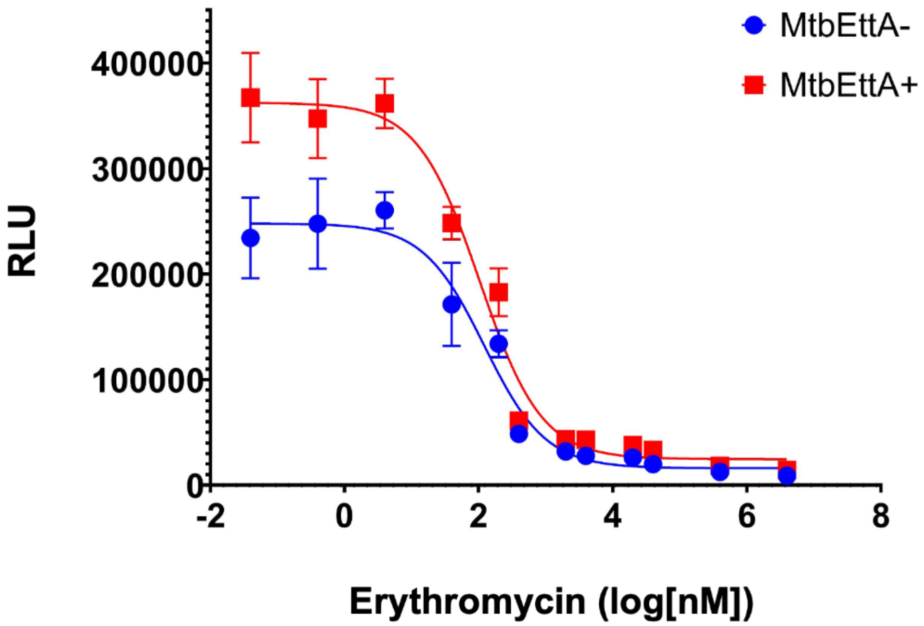

Supplementary Fig. 1. The effect of MtbEttA on the IC50 of erythromycin examined using an *in-vitro* translation assay. Relative light units (RLU) are recorded according to the production of nanoluciferase in the *in-vitro* translation system. A series of erythromycin concentrations (0.04nM-4mM) is used with (red) or without (blue) MtbEttA to determine the IC50 values, which are around 104nM and 122nM with and without MtbEttA, respectively. N=2 independent measurements are performed. Data are presented as mean values +/- SD. Source data are provided as a Source Data file.

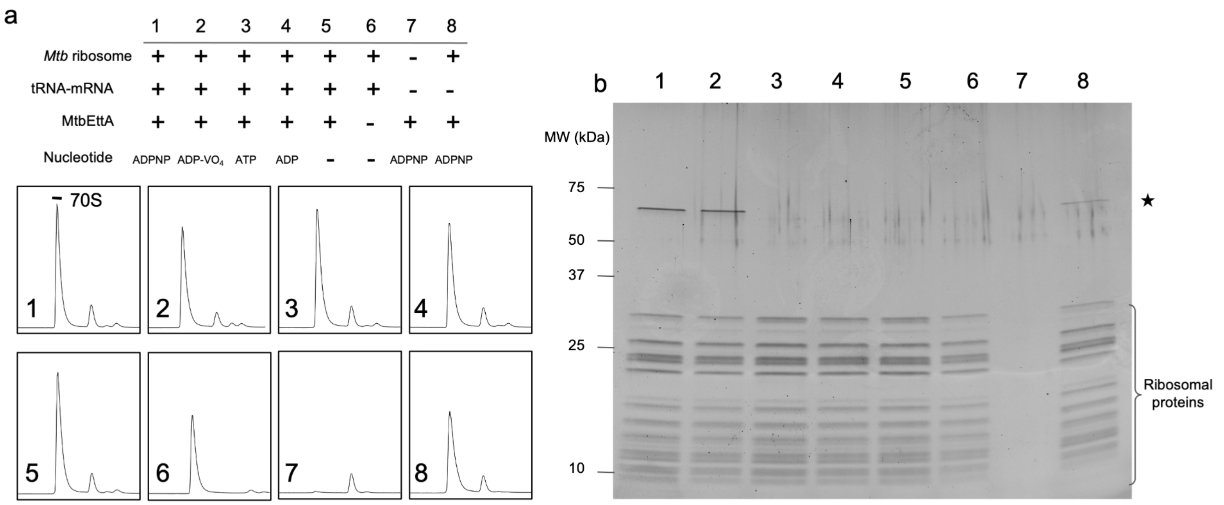

Supplementary Fig. 2. Assessments of the conditions for forming a stable MtbEttA-ribosome complex. **a**, Experiment conditions are listed in the table, with "+" and "–" as included and excluded, respectively. Chromatogram for each condition is shown in the box, with the 70S peak labelled as in Panel 1. **b**, SDS-PAGE showing the composition of 70S peak in corresponding conditions. The gel is stained by SYPRO^{TM} Ruby. MtbEttA is indicated by the star. More than three times of each experiment were repeated independently with similar results.

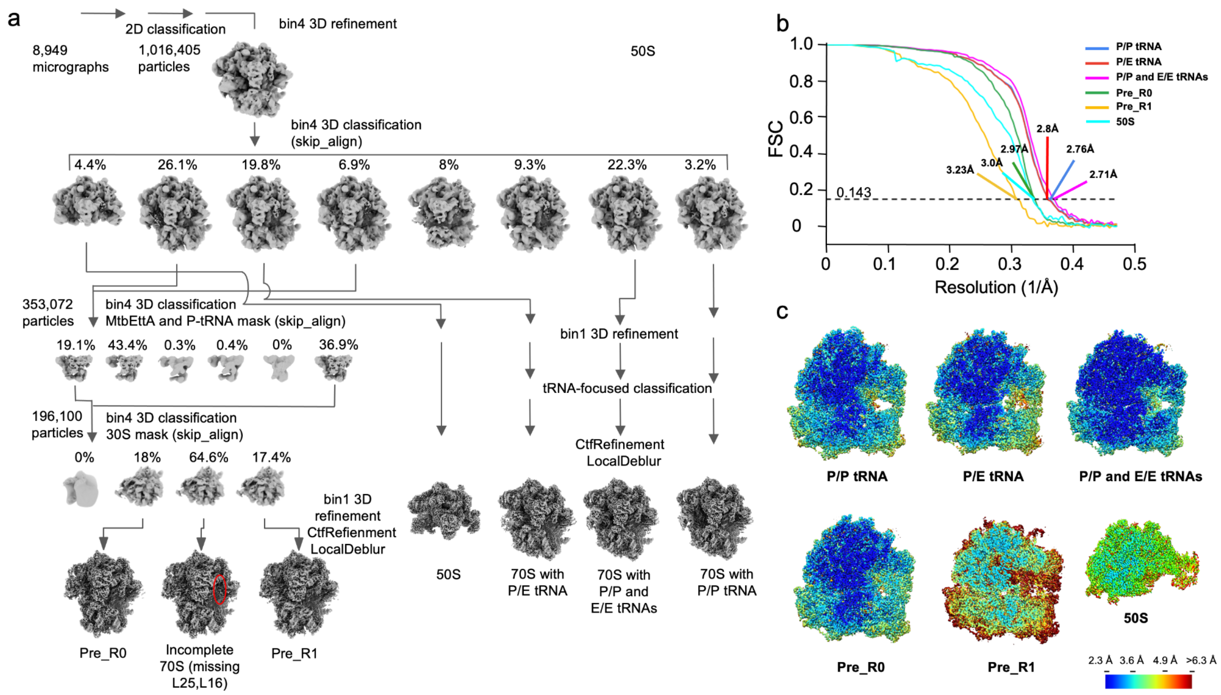

Supplementary Fig. 3. The cryo-EM data processing workflow for 70SIC-MtbEttA with ADPNP. **a**, Single-particle data processing strategy for 70SIC-MtbEttA with ADPNP. Red oval indicates the missing of bL25 and uL16. **b**, FSC curves of the reconstructed cryo-EM maps. Gold-standard was used to estimate the resolution (FSC=0.143). **c**, Cutaway view of the cryo-EM maps, which are colored by local resolutions.

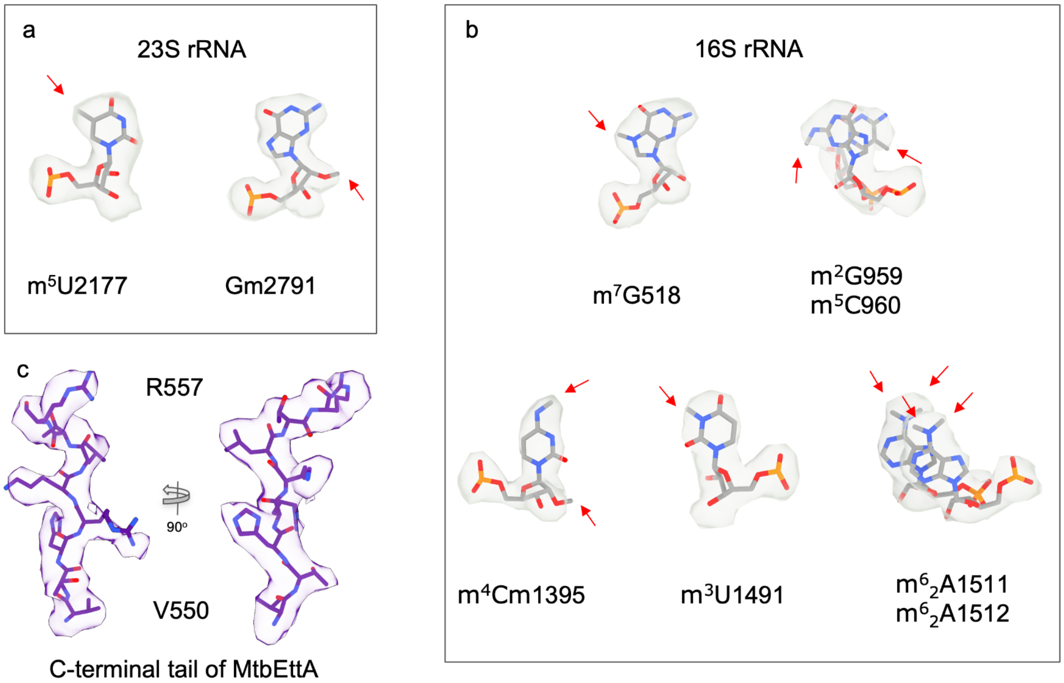

Supplementary Fig. 4. Examples of the cryo-EM density. a,**b**, RNA modifications in ribosomal 23S rRNA and 16S rRNA in the *Mtb* 70S with P/P and E/E tRNAs. Cryo-EM density is shown in transparent light gray and modification sites are labelled with red arrows. **c**, Cryo-EM density and model of the C-terminal tail of MtbEttA in the Pre_R0 state.

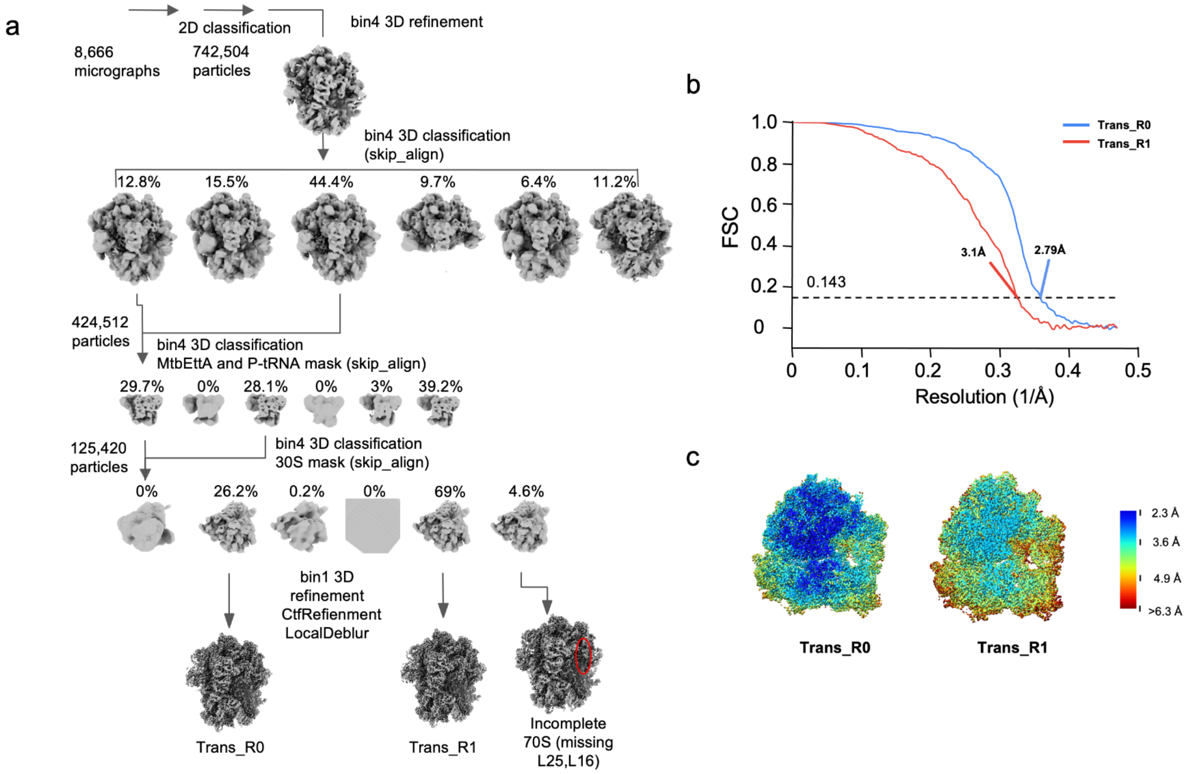

Supplementary Fig. 5. Cryo-EM data processing workflow for 70SIC-MtbEttA with ADP-VO_4. a, Single-particle data processing strategy for 70SIC-MtbEttA with ADP-VO_4. Red oval indicates the missing of bL25 and uL16. **b**, FSC curves of the reconstructed cryo-EM maps. Gold-standard was used to estimate the resolution (FSC=0.143). **c**, Cutaway view of the cryo-EM maps, which are colored by local resolutions. The MtbEttA-free states were not further processed due to their high similarity to the corresponding ones observed in the ADPNP states.

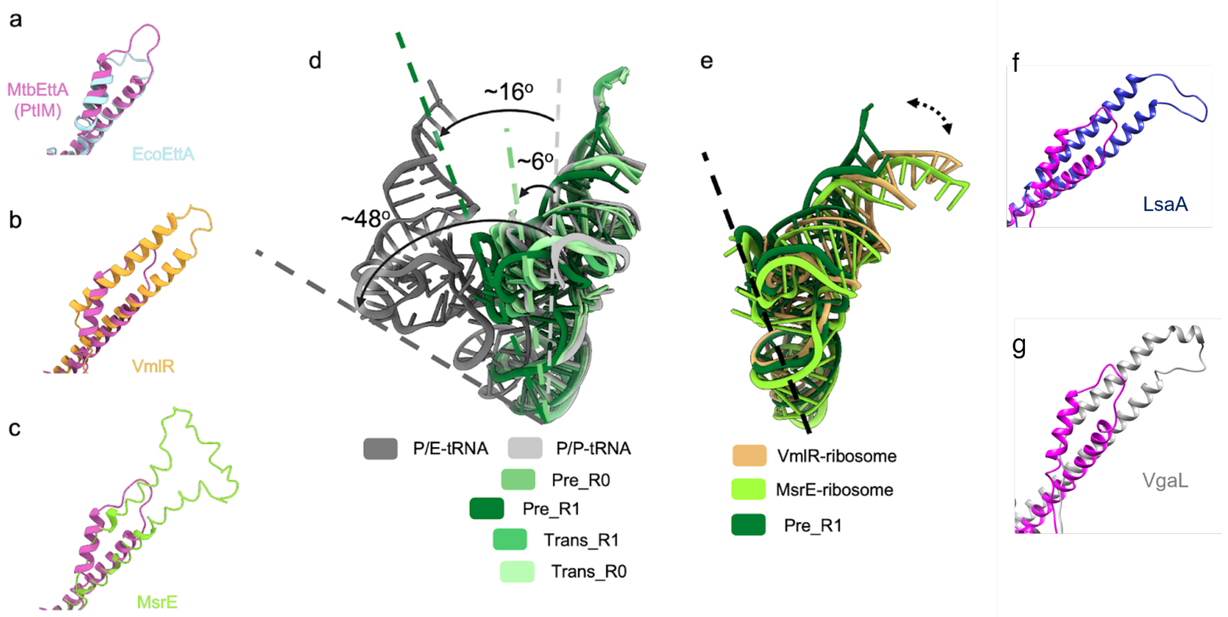

Supplementary Fig. 6. Conformational comparisons of PtIM and tRNA among MtbEttA, EcoEttA, VmlR, and MsrE. a,**b**,**c**, Comparison of the PtIM of MtbEttA in the Pre_R0 state with EcoEttA (**a**), VmlR (**b**), MsrE (**c**), LsaA (**f**) and VgaL (**g**)[1]. **d**, tRNA positions in Pre_R0, Pre_R1, Trans_R0, and Trans_R1 states, compared to P/P-tRNA and P/E-tRNA. Dashed lines indicate the elbow region of the tRNA. The direction and degree of the movement of the tRNA elbow in the Pre_R0 and Pre_R1 states relative to P/P-tRNA position are labelled by arrows. **e**, Comparison of tRNA positions in the Pre_R1 state of MtbEttA-70SIC, VmlR-ribosome and MsrE-ribosome complexes. Dashed line and double-headed arrow indicate the elbow region and the CCA tail of the tRNA, respectively.

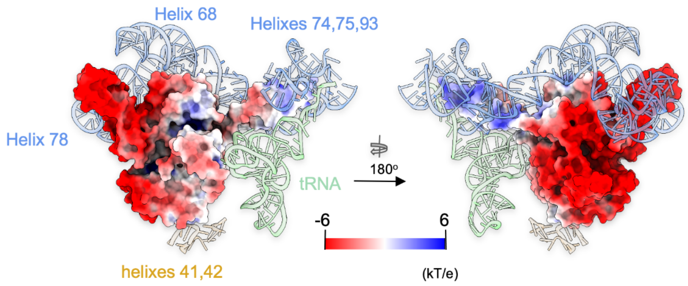

Supplementary Fig. 7. The electrostatic surface potential of MtbEttA. MtbEttA surface is colored by the electrostatic potential as indicated by the color scale bar. Ribosomal RNA Helices 68, 74-75, 78, 93 of 23S rRNA and the tRNA are shown as transparent ribbon models.

Supplementary Fig. 8. Sequence alignment of nine representative EttA-like proteins from different species and the conservation of the key residues. a, Nine different EttA-like proteins (Arabidopsis ABCF5 and Arabidopsis ABCF2 from *Arabidopsis thaliana*, HaemoEttA from *Haemophilus influenzae*, ChvD from *Agrobacterium tumefaciens*, UuP from *Buchnera aphidicola*, HumanABCF3 from *Homo sapiens*, YdiF from *Bacillus subtilis*, EcoEttA from *Escherichia coli*) were chosen after BLASTP (E value < e^-50). Then the amino acid sequences are aligned based on evolutionary conservation using Consurf Server[2,3] (Cyan: variable, Magenta: conserved). Key motifs are marked. **b,** MtbEttA structure colored based on amino acid sequence conservation (Red: variable, Blue: conserved).

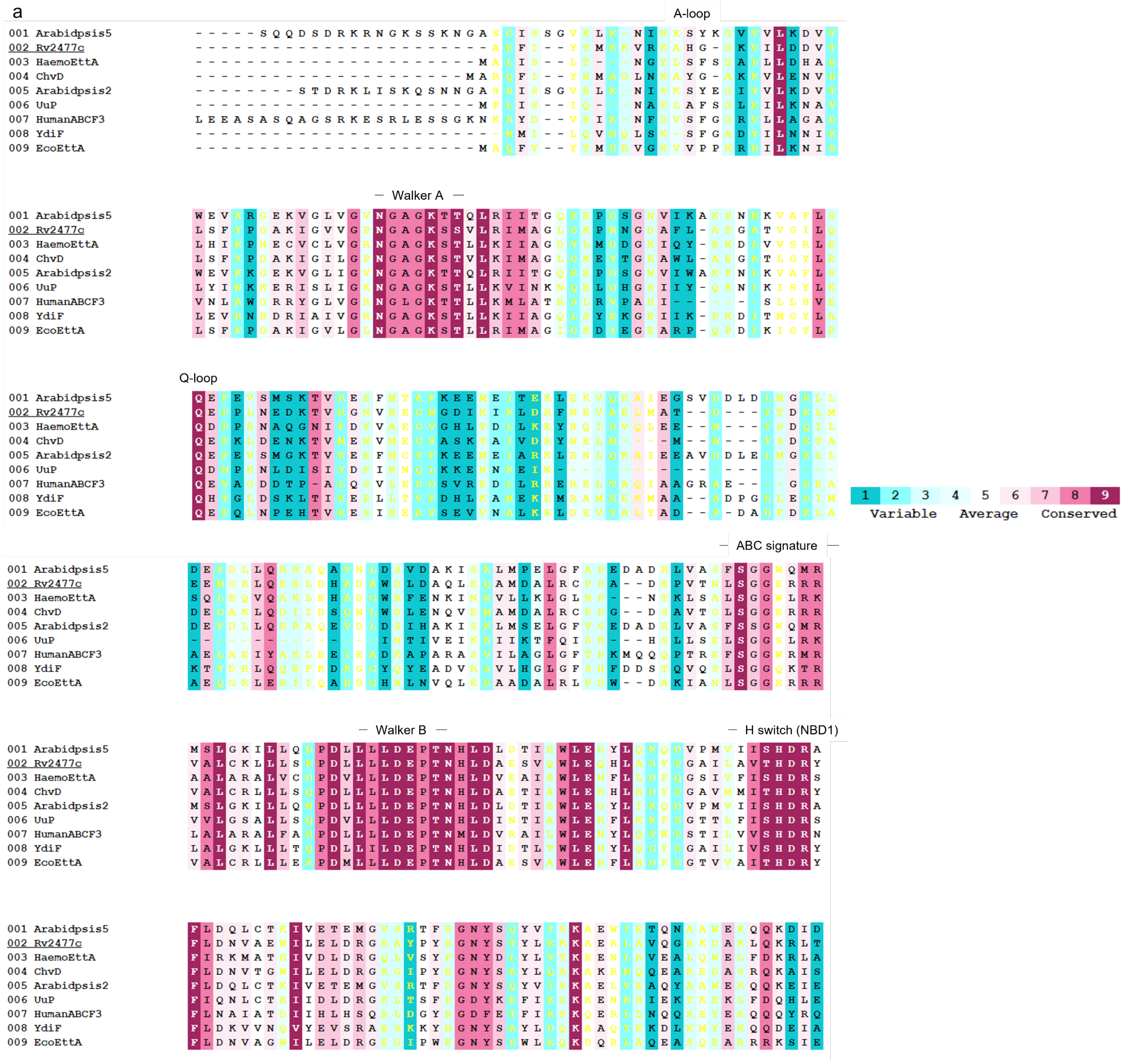

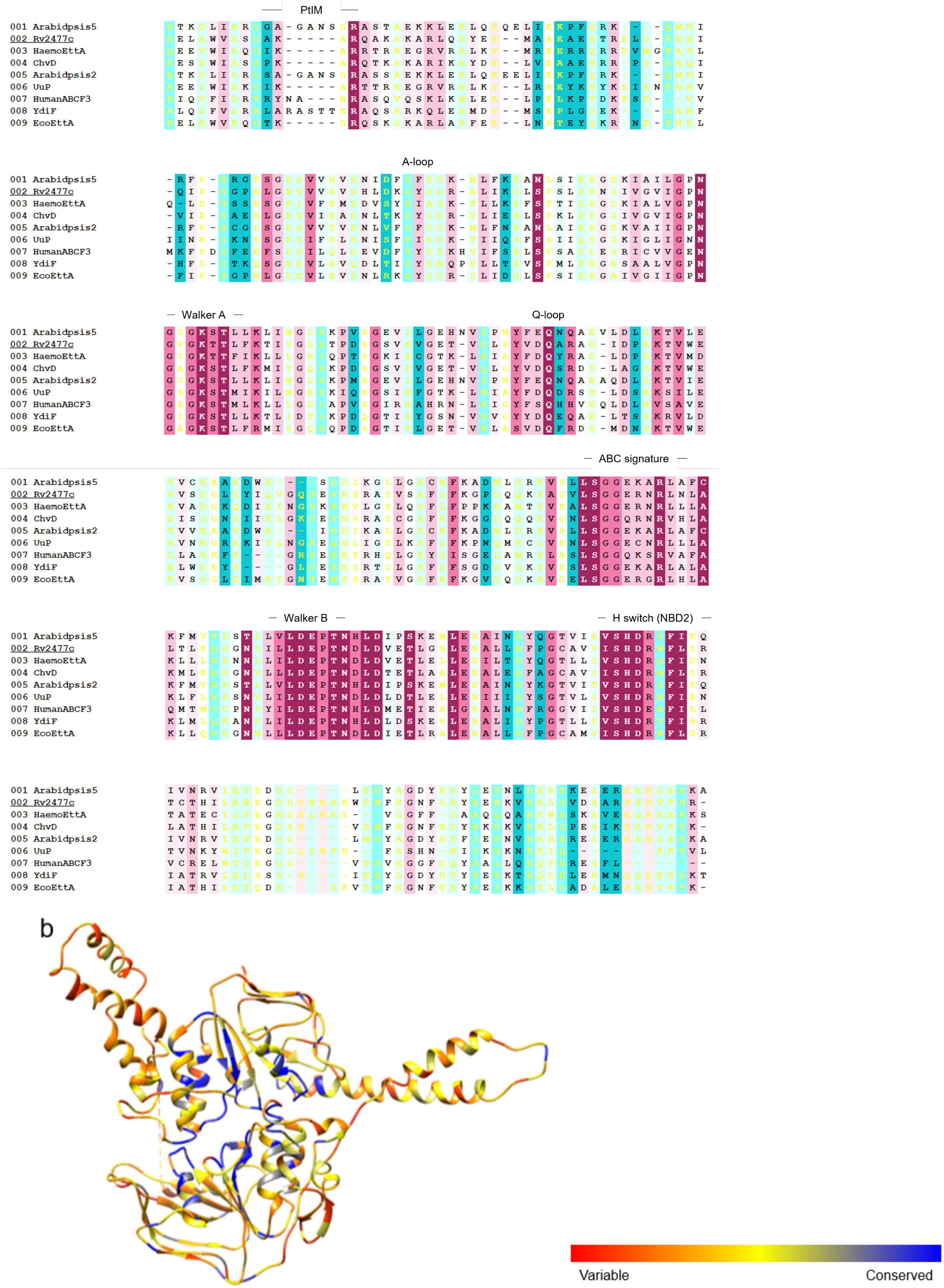
PtIM
A-loop
Walker A
Q-loop
ABC signature
Walker B
H switch (NBD2)
001 Arabidpsis5
002 Rv2477c
003 HaemoEttA
004 ChvD
005 Arabidpsis2
006 UuP
007 HumanABCF3
008 YdiF
009 EcoEttA
b
Variable
Conserved

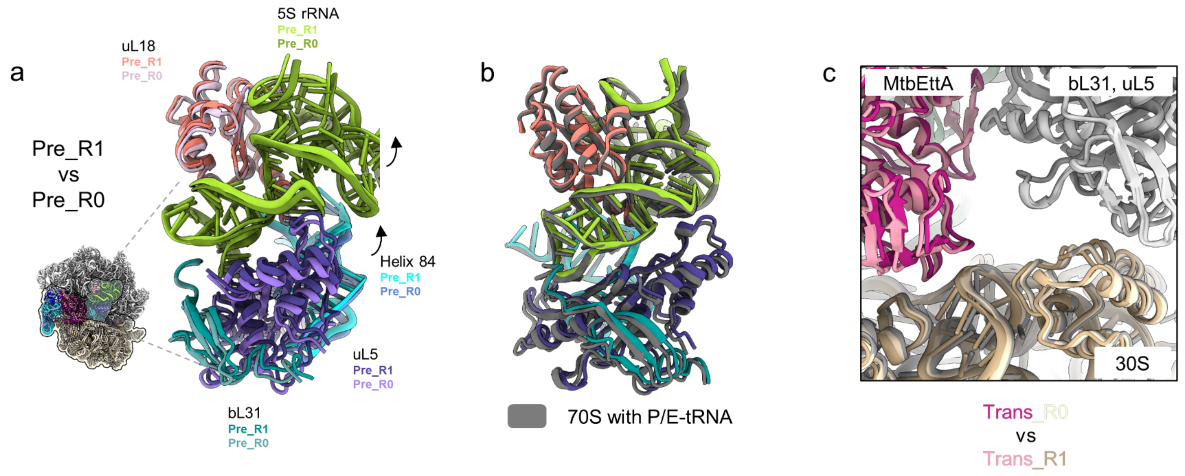

Supplementary Fig. 9. Remodeling of the 50S pre-hydrolysis and transition states. a, Structural comparison between the Pre_R0 and Pre_R1 states. Models of 70SIC-MtbEttA complex at the Pre_R0 and Pre_R1 states were superimposed based on the 23S rRNA. Outlines on the ribosome model label two structural regions, which show opposite directions of movement. MtbEttA, tRNA, handle and L1 stalk move in the same direction as the 30S rotates. Proteins bL31, uL5, uL18 and 5S rRNA move in the opposite direction as the 30S rotates. The close-up view shows the difference between the Pre_R0 and Pre_R1 states around the 5S rRNA. **b**, Overlap of the Pre_R1 state (colored) and the 70S with P/E-tRNA (dark gray). **c**, Comparison of Trans_R0 and Trans_R1 states in the same view as in **Fig. 3b**. The 30S subunit of *Mtb* 70S with P/P-tRNA is colored in light gray. No obvious movement is observed for proteins bL31 and uL5, which are colored in light gray.

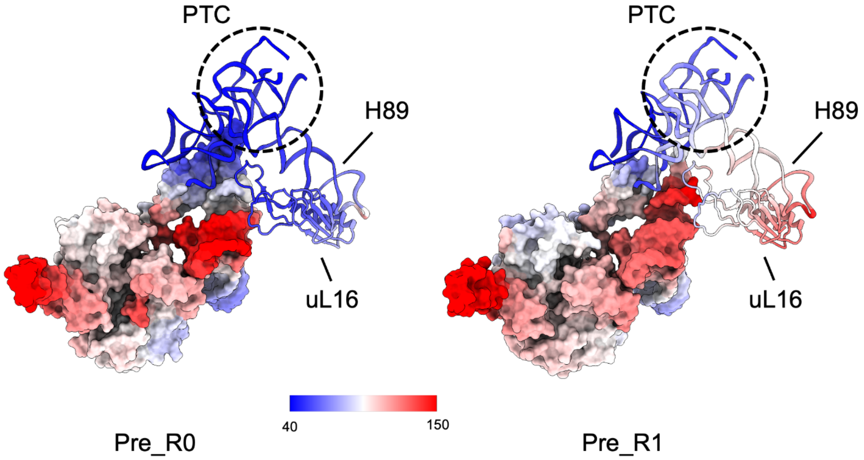

Supplementary Fig. 10. B-factor colored on the models of MtbEttA, tRNA, PTC and uL16 in the Pre_R0 and Pre_R1 states. MtbEttA and tRNA are shown in surface representation. PTC (G2290-U2312, A2668-C2750 and G2812-C2855) and uL16 are shown as ribbon models.

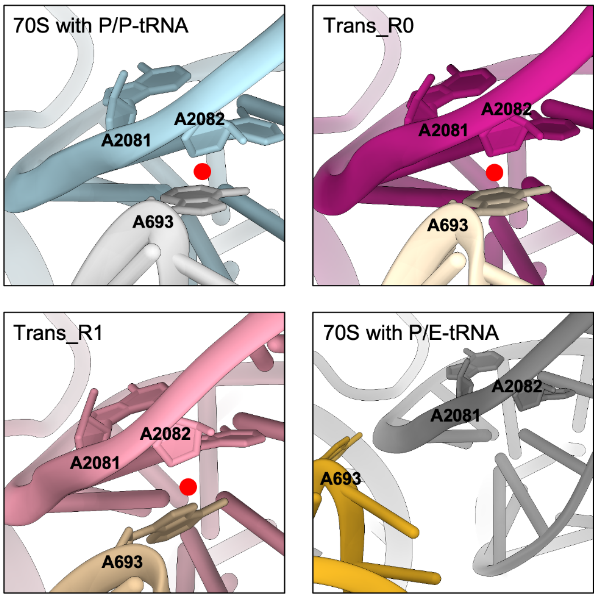

Supplementary Fig. 11. Intersubunit bridge B7a in the *Mtb* 70S with P/P-tRNA, *Mtb* 70S with P/E-tRNA, 70SIC-MtbEttA complexes at the Trans_R0 and Trans_R1 states are shown as in **Fig. 3c**. Red dot indicates the nucleotide base stacking interaction.

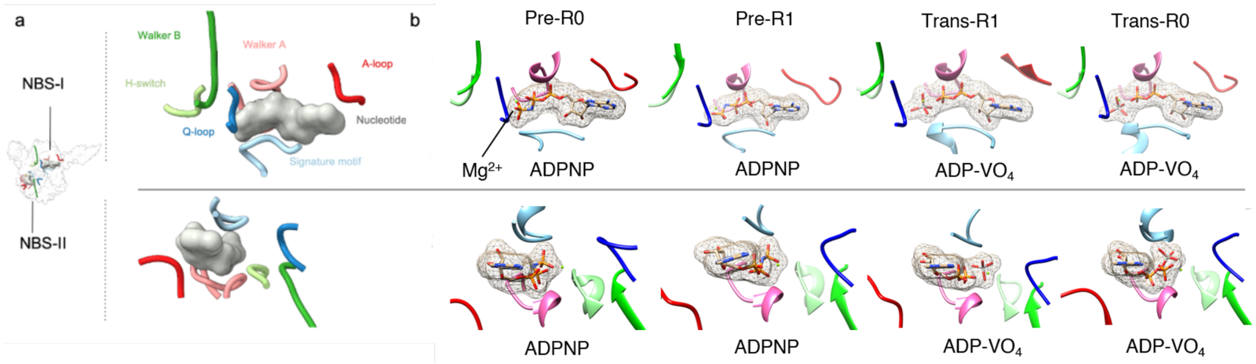

Supplementary Fig. 12. Cryo-EM density of ATP analogues in the nucleotide binding sites (NBSs). a, Zoom-in view of conserved motifs in the NBSs. Nucleotides are shown as gray molecular surfaces. **b**, Cryo-EM densities of ADPNP and ADP-VO_4 are shown as mesh surfaces. Mg^{2+} molecules are shown as green spheres.

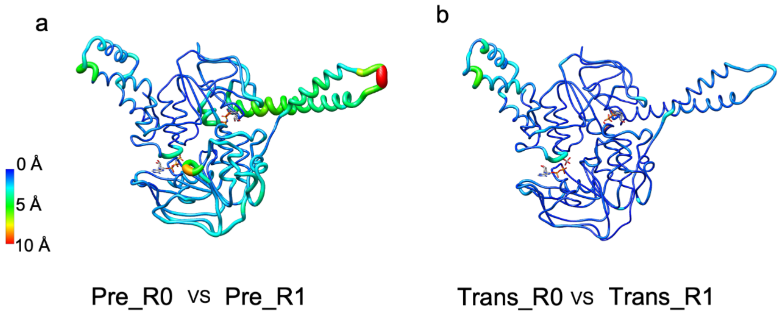

Supplementary Fig. 13. Structural plasticity of MtbEttA in the pre-hydrolysis and transition states. a,**b**, Tube representations of Cα distances between the Pre_R0 and Pre_R1 states in **a**, between the Trans_R0 and Trans_R1 in **b**. Red color and wider tubes indicate larger Cα distances. MtbEttA shows larger conformational differences in pre-hydrolysis states.

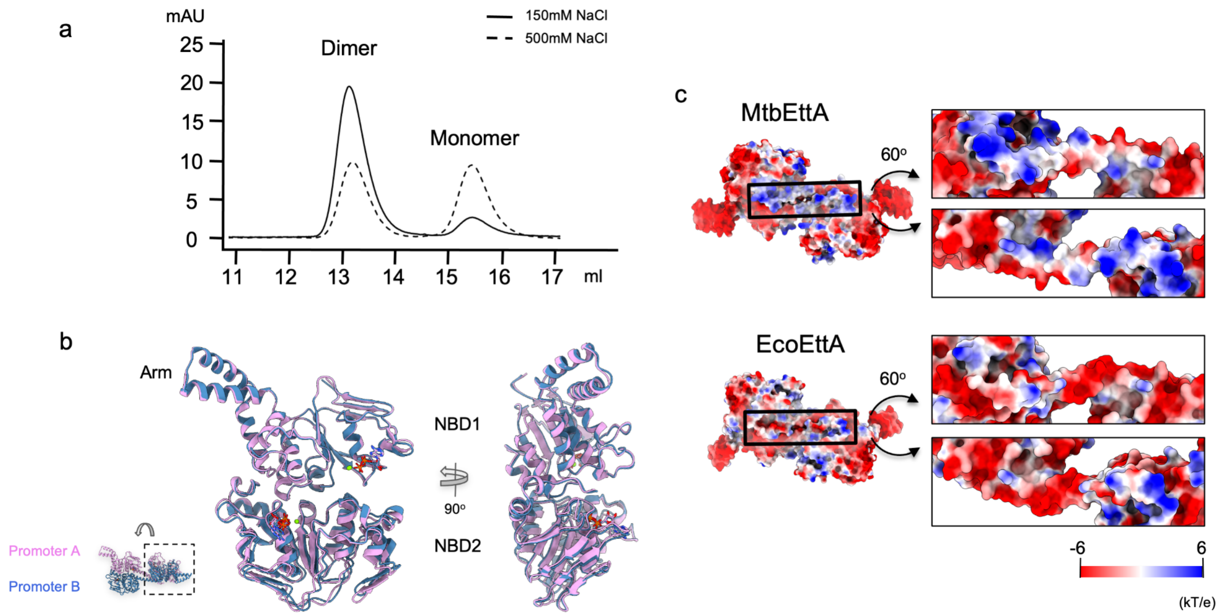

Supplementary Fig. 14. The dimer/monomer transition of MtbEttA and its electrostatic surface compared with EcoEttA. a, Size exclusion chromatography of apo MtbEttA at 10μM concentration. Different sodium chloride concentrations are plotted in different line styles. **b**, Similar domain organization between two halves of the domain-swapped dimer of MtbEttA with ADP molecules. The second half outlined by dashed square (residues 306-550 in protomer A and 2-241 in protomer B) is superimposed to the first half (residues 2-241 in protomer A and 306-550 in protomer B) based on NBD1. **c**. Surfaces of MtbEttA and EcoEttA dimers are colored according to the electrostatic potential. The insets show the electrostatic potential of PtIM from the two protomers.

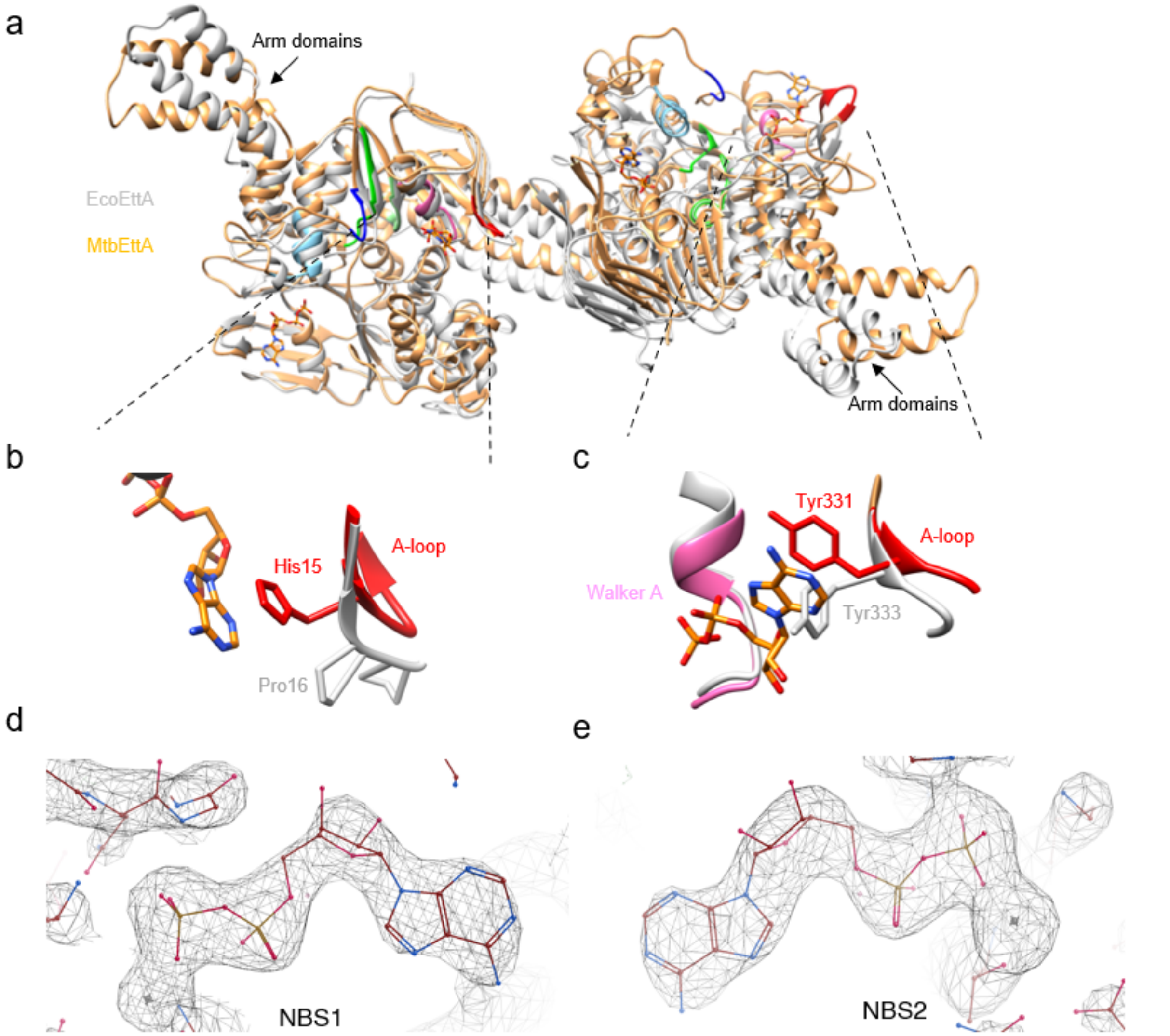

Supplementary Fig. 15. The structural comparison between the crystal structures of ADP-bound MtbEttA and nucleotide-free EcoEttA. a, Overall structure alignment of ADP-bound MtbEttA (gold) and nucleotide-free EcoEttA (gray, PDB: 4FIN). **b,** In NBS1, His15 of MtbEttA in the A-loop forms a stacking interaction with an adenine ring of ADP[4] whereas Pro16 of EcoEttA is unlikely to reach the adenine ring in this conformation. **c**, In NBS2, Tyr331 of MtbEttA in the A-loop forms a stacking interaction with the adenine ring of ADP whereas the corresponding Tyr333 in the nucleotide-free EcoEttA will collide with the ADP. **d**,**e**, Electron densities of the ADP and magnesium ions in the crystal structure of the ADP-bound MtbEttA in both NBSs.

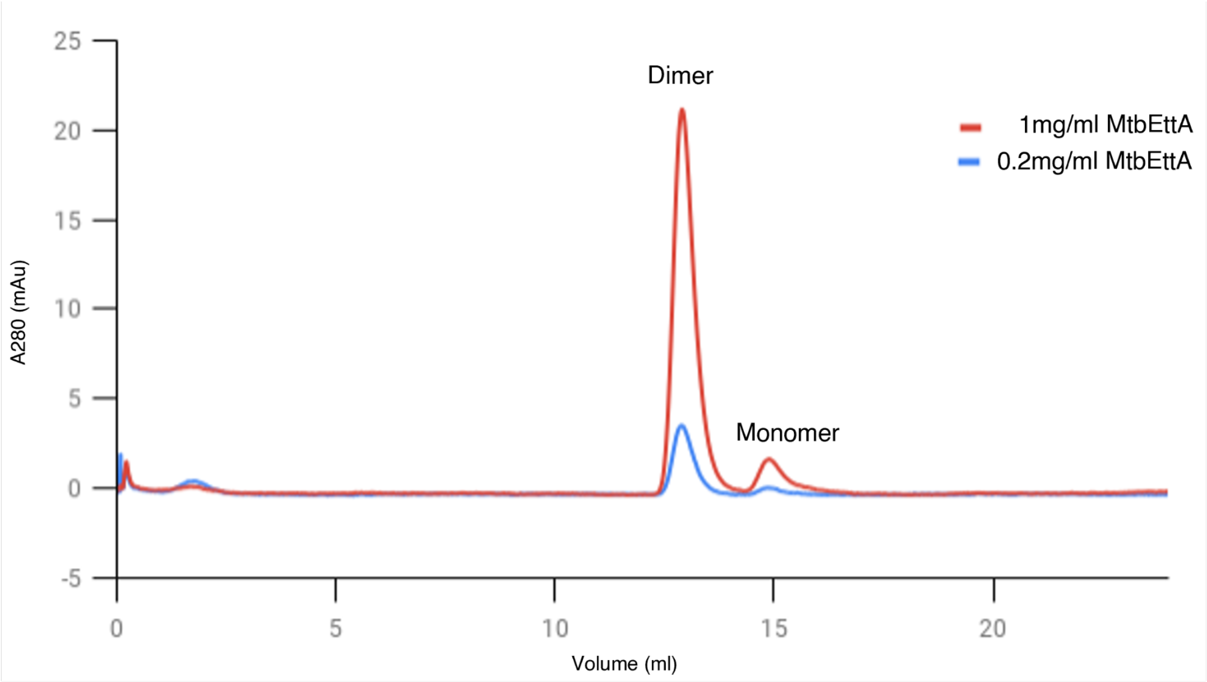

Supplementary Fig. 16. Chromatogram of MtbEttA at different concentrations. Based on the area under the curve of absorbance peaks, the ratio of dimer:monomer is around 8:1 at 1mg/ml (or ~16μM) concentration, whereas the ratio of dimer:monomer is still around 6:1 at 0.2mg/ml (or ~3μM) concentration.

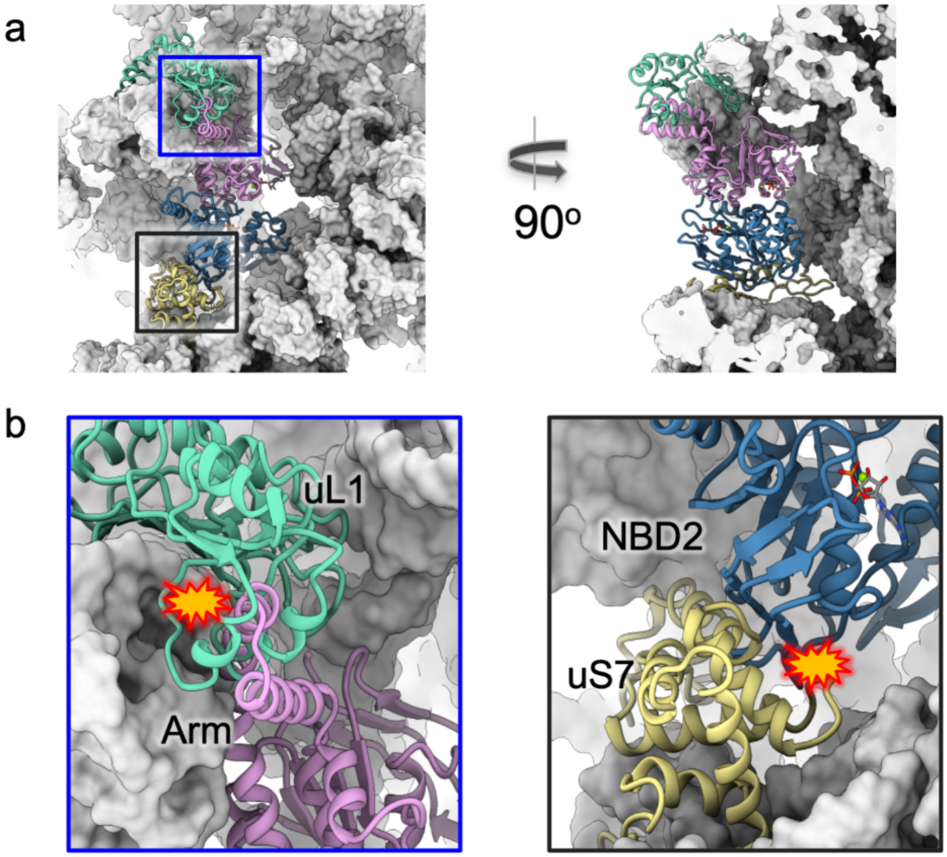

Supplementary Fig. 17. Potential steric clashes between the Mtb 70SIC and MtbEttA in the post-hydrolysis state. a, MtbEttA in the post-hydrolysis state is superimposed with MtbEttA in Trans_R0 state based on NBD1. *Mtb* ribosome is shown as a light gray surface. uL1 and uS7 are shown as ribbon models and colored in green and yellow, respectively. **b**, The close-up views showing the clashes, indicated by orange explosion signs, between uL1 and the Arm domain, uS7 and NBD2, respectively.

Supplementary Table 1. Data collection and refinement statistics (molecular replacement) of the crystal structure of MtbEttA in the ADP state and the Ramachandran plot.

	MtbEttA-ADP
**Data collection**	
Space group	P 2 21 21
Cell dimensions	
a, *b*, *c* (Å)	50.06, 106.96, 269.19
α, β, γ (°)	90.00, 90.00, 90.00
Resolution (Å)	49.22-2.9 (3.004-2.9)
R_{sym} or R_{merge}	0.0525 (0.4825)
$I / \sigma I$	8.16 (1.85)
Completeness (%)	99.81 (99.72)
Redundancy	2.0 (2.0)
**Refinement**	
Resolution (Å)	49.22-2.9 (3.004-2.9)
No. reflections(total/unique)	65870/33131
R_{work} / R_{free} (%)	20.39/24.51
No. atoms	8685
Protein	8573
Ligand/ion	112
Water	0
B-factors	
Protein	77.45
Ligand/ion	92.07
R.m.s. deviations	
Bond lengths (Å)	0.003
Bond angles (°)	0.74

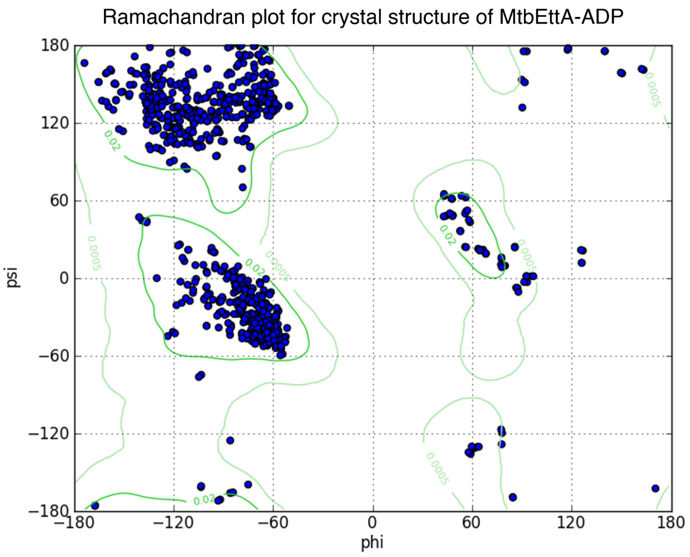

Supplementary References:

1. Crowe-McAuliffe, C. et al. Structural basis of ABCF-mediated resistance to pleuromutilin, lincosamide, and streptogramin A antibiotics in Gram-positive pathogens. *Nat Commun* **12**, 3577 (2021).
2. Celniker, G. et al. ConSurf: Using Evolutionary Data to Raise Testable Hypotheses about Protein Function. *Isr J Chem* **53**, 199-206 (2013).
3. Ashkenazy, H., Erez, E., Martz, E., Pupko, T. & Ben-Tal, N. ConSurf 2010: calculating evolutionary conservation in sequence and structure of proteins and nucleic acids. *Nucleic Acids Research* **38**, W529-W533 (2010).
4. Luscombe, N. M., Laskowski, R. A. & Thornton, J. M. Amino acid-base interactions: a three-dimensional analysis of protein-DNA interactions at an atomic level. *Nucleic Acids Res* **29**, 2860-2874 (2001).
