# Supplementary material for: Interplay between an ATP-binding cassette F protein and the ribosome from Mycobacterium tuberculosis
Source: Nat Commun. 2022 Jan 21;13:432. doi: 10.1038/s41467-022-28078-1 (PMC8782954; doi:10.1038/s41467-022-28078-1)
Supplement: Supplementary file 3 — Description of Additional Supplementary Files [file 41467_2022_28078_MOESM3_ESM.pdf]

Description of Additional Supplementary Files

File Name: Supplementary Movie 1
Description: Morphing between the two structures of 70SIC-MtbEttA complex in the pre-hydrolysis states.

File Name: Supplementary Movie 2
Description: Asymmetric nucleotide-engagement in the two nucleotide-binding sites of MtbEttA in the pre-hydrolysis states.

File Name: Supplementary Movie 3
Description: Morphing between the structures of MtbEttA in the Trans_R0 and post-hydrolysis states.
