# Supplementary material for: Interplay between an ATP-binding cassette F protein and the ribosome from Mycobacterium tuberculosis
Source: Nat Commun. 2022 Jan 21;13:432. doi: 10.1038/s41467-022-28078-1 (PMC8782954; doi:10.1038/s41467-022-28078-1)
Supplement: Supplementary file 7 — Reporting Summary [file 41467_2022_28078_MOESM7_ESM.pdf]

nature portfolio

Corresponding author(s): Junjie Zhang

Last updated by author(s): Dec 24, 2021

Reporting Summary

Nature Portfolio wishes to improve the reproducibility of the work that we publish. This form provides structure for consistency and transparency in reporting. For further information on Nature Portfolio policies, see our Editorial Policies and the Editorial Policy Checklist.

Statistics

For all statistical analyses, confirm that the following items are present in the figure legend, table legend, main text, or Methods section.

n/a	Confirmed	
[ ]	[x]	The exact sample size (*n*) for each experimental group/condition, given as a discrete number and unit of measurement
[ ]	[x]	A statement on whether measurements were taken from distinct samples or whether the same sample was measured repeatedly
[x]	[ ]	The statistical test(s) used AND whether they are one- or two-sided<br>*Only common tests should be described solely by name; describe more complex techniques in the Methods section.*
[x]	[ ]	A description of all covariates tested
[x]	[ ]	A description of any assumptions or corrections, such as tests of normality and adjustment for multiple comparisons
[ ]	[x]	A full description of the statistical parameters including central tendency (e.g. means) or other basic estimates (e.g. regression coefficient) AND variation (e.g. standard deviation) or associated estimates of uncertainty (e.g. confidence intervals)
[x]	[ ]	For null hypothesis testing, the test statistic (e.g. *F*, *t*, *r*) with confidence intervals, effect sizes, degrees of freedom and *P* value noted<br>*Give P values as exact values whenever suitable.*
[x]	[ ]	For Bayesian analysis, information on the choice of priors and Markov chain Monte Carlo settings
[x]	[ ]	For hierarchical and complex designs, identification of the appropriate level for tests and full reporting of outcomes
[x]	[ ]	Estimates of effect sizes (e.g. Cohen's *d*, Pearson's *r*), indicating how they were calculated

Our web collection on statistics for biologists contains articles on many of the points above.

Software and code

Policy information about availability of computer code

Data collection	EPU 2.
Data analysis	Relion 3, Coot 0.8, Chimera 1.14, ChimeraX 1.2, ISOLDE 1.2, Phenix dev 2306.

For manuscripts utilizing custom algorithms or software that are central to the research but not yet described in published literature, software must be made available to editors and reviewers. We strongly encourage code deposition in a community repository (e.g. GitHub). See the Nature Portfolio guidelines for submitting code & software for further information.

Data

Policy information about availability of data

All manuscripts must include a data availability statement. This statement should provide the following information, where applicable:

- Accession codes, unique identifiers, or web links for publicly available datasets
- A description of any restrictions on data availability
- For clinical datasets or third party data, please ensure that the statement adheres to our policy

The cryo-EM maps and models of Mtb 70SIC with MtbEttA at Pre_R0, Pre_R1, Trans_R0, Trans_R1 states and Mtb 70S with P/P tRNA, P/E tRNA, P/P and E/E tRNAs, are deposited in the EMData Bank with accession IDs: EMD-23961, EMD-23962, EMD-23969, EMD-23972, EMD-23974, EMD-23975, and EMD-23976, and in the Protein Data Bank with accession IDs, 7MSC, 7MSH, 7MSM, 7MSZ, 7MT2, 7MT3 and 7MT7, respectively. The cryo-EM map of Mtb 50S is deposited in the EMData Bank with accession ID EMD-23981. The model of ADP-bound MtbEttA is deposited in the Protein Data Bank with accession ID 7MU0. The previous structures of the Mtb 70S ribosome and EcoEttA, which facilitated our modeling, can be accessed from the Protein Data Bank with accession IDs 5V93 and 4FIN, respectively.

Field-specific reporting

Please select the one below that is the best fit for your research. If you are not sure, read the appropriate sections before making your selection.

- [x] Life sciences
- [ ] Behavioural & social sciences
- [ ] Ecological, evolutionary & environmental sciences

For a reference copy of the document with all sections, see nature.com/documents/nr-reporting-summary-flat.pdf

Life sciences study design

All studies must disclose on these points even when the disclosure is negative.

Sample size	In total, we have 1,175,176 particles selected from 8,949 micrographs for the 70SIC-MtbEttA-ADPNP sample and 742,504 particles selected from 8,666 micrographs for the 70SIC-MtbEttA-ADPNP sample to achieve atomic resolutions. These are described in Extended Data Fig.3 and 5. Such a data size were chosen to allow us resolved the atomic details of the structures, particularly for reliably distinguishing the nucleotides in the NBSs of MtbEttA.
Data exclusions	All the data were included in the analysis following the criteria set by Relion.
Replication	Given the large dataset needed to solve a cryo-EM structure to high-resolution, we only collected one dataset for each state without replication. However, during resolution assessment, each dataset was separated into two halves to calculate the Fourier Shell Correlation. In vitro translation assays are representative of two independent experiments.
Randomization	For Cryo-EM, particle images are randomly picked for the analysis; division of datasets into two random halves was done based on standard approach in RELION 3. Other experiments did not involve randomization.
Blinding	Blinding was not applicable to this study because this type of study does not use group allocation.

Reporting for specific materials, systems and methods

We require information from authors about some types of materials, experimental systems and methods used in many studies. Here, indicate whether each material, system or method listed is relevant to your study. If you are not sure if a list item applies to your research, read the appropriate section before selecting a response.

Materials & experimental systems

n/a	Involved in the study
x	Antibodies
x	Eukaryotic cell lines
x	Palaeontology and archaeology
x	Animals and other organisms
x	Human research participants
x	Clinical data
x	Dual use research of concern

Methods

n/a	Involved in the study
x	ChIP-seq
x	Flow cytometry
x	MRI-based neuroimaging
